# Assessing individual differences in grouping strategy in visual working memory

**DOI:** 10.3758/s13414-025-03013-w

**Published:** 2025-02-26

**Authors:** Yin-ting Lin, Andrew B. Leber

**Affiliations:** Department of Psychology, The Ohio State University, 225 Psychology Building, 1835 Neil Avenue, Columbus, OH 43210 USA

**Keywords:** Individual differences, Working memory capacity, Strategy use

## Abstract

Visual working memory is highly limited. Fortunately, we can use various strategies to improve performance. For example, we can make use of grouping cues, such as an illusory shape (Kanizsa figure), to optimize encoding and storage of information. However, it remains unclear how strategy use varies across individuals. In the current study, we examined whether working memory ability is related to individual differences in the use of grouping strategy. We manipulated whether the items formed Kanizsa figures or were randomly oriented in an orientation change detection task. We explicitly informed participants of the manipulation. Moreover, we independently assessed visual working memory capacity in a colour change detection task. In Experiment 1, there was better memory performance for Kanizsa figures versus randomly oriented items. Importantly, we found that individuals with higher working memory capacity showed a larger Kanizsa benefit. In Experiment 2, we still found a memory benefit for Kanizsa figures when we sequentially presented the inducers. This suggests that participants are able to use more abstract rules to strategically encode items. Furthermore, we again found that high working memory capacity corresponds to a larger Kanizsa benefit, although this finding was less robust across multiple analysis methods. Taken together, these findings show that individual differences in working memory ability predict effective use of grouping strategy. Moreover, these individual differences may partly reflect grouping based on high-level knowledge.

**Supplementary information:**

The online version contains supplementary material available at 10.3758/s13414-025-03013-w.

## Introduction

Working memory (WM) allows us to maintain information for short periods of time. However, we can only hold limited information at a given time. Fortunately, we can use a number of strategies to boost WM performance (see Gonthier, 2021, for a review). One common way of improving performance is to use a grouping or chunking strategy. Studies have shown improved memory for items grouped by feature similarity (Morey et al., 2015; Peterson & Berryhill, 2013; Prieto et al., 2021), illusory shapes (Allon et al., 2019; Chen et al., 2021; Diaz et al., 2021), common fate (Luria & Vogel, 2014), and proximity (Woodman et al., 2003; Xu, 2006). Interestingly, beyond perceptual grouping effects (e.g., based on Gestalt principles), more conceptual forms of grouping have been shown to benefit WM. For instance, people can make use of structural regularities even when items are sequentially presented (Gao et al., 2016; Zhang & Du, 2022). These findings broadly suggest that we can optimize encoding and storage of information by organizing information into meaningful units.

Although there is ample evidence showing that strategies improve WM, less is known about how strategy use varies across individuals. There has been considerable interest in understanding individual differences in WM. Yet, much of the work on individual differences examined the degree to which WM performance relates to storage capacity and attentional control abilities (Cowan et al., 2005; Fukuda et al., 2015; Unsworth et al., 2020). Importantly, it has been proposed that individual differences in WM may reflect differences in both ability and strategy (Gonthier, 2021; Pearson & Keogh, 2019). One possibility is that individuals with high WM ability tend to use more effective strategies (Lin & Leber, 2024; Linke et al., 2011). An alternative view proposes that individuals with low WM ability may be more likely to use strategies to compensate for their weaknesses (e.g., Castel et al., 2013). Yet another possibility is that individual differences in ability and strategy provide independent contributions to WM performance.

The current study attempts to test the three competing accounts of how WM ability relates to the use of grouping strategy. We used a change detection task to assess orientation memory for items that either formed Kanizsa figures (illusory shapes) or had random orientations. Previous work using this method has shown better performance with Kanizsa figures, which are thought to reduce memory demands (Diaz et al., 2021; McCollough, 2011). We adopt this method in Experiment 1, and we measure how the Kanizsa advantage varies as a function of independently-assessed WM capacity. In Experiment 2, we sequentially presented items that form Kanizsa figures, thus removing the illusory phenomenon, to further examine whether individuals use grouping strategies based on more abstract rules.

## Experiment 1

### Method

#### Participants

Fifty participants (25 women, 25 men; mean age = 19.52 years, age range =18 - 26) completed a 1-hr experiment for course credit or payment of $10/hr. Four participants were excluded and replaced due to negative *K* values or performance not reliably above chance in the orientation memory task (below 54.67%, cutoff determined by the binomial cumulative distribution function).

All participants reported normal vision or corrected-to-normal vision and normal colour vision. Experiments were approved by The Ohio State University Institutional Review Board, and all participants provided written informed consent.

We chose our sample size based on a “rule of thumb” of at least N=50 to examine relationships among two variables (Wilson Van Voorhis & Morgan, 2007). A sensitivity power analysis in G*Power showed that this sample size provides 80% power for detecting a correlation of *r* = .37 (Faul et al., 2007).

#### Apparatus

The study was run on a Mac Mini computer and a 23-inch Acer G235H monitor (60 Hz refresh rate, 1,920 × 1,080 pixels). Participants were seated approximately 60 cm from the monitor. All experiments were programmed in MATLAB and Psychtoolbox (Kleiner et al., 2007). Stimuli were presented against a grey background.

#### Orientation memory task

Each memory display consisted of six “pacman” stimuli (white circles with a 60° triangular gap, radius 1°). Stimuli were presented within a 20° × 20° window around central fixation, with a minimum distance of 2° from fixation. The stimuli were arranged in two triangular configurations (each side 3.6°) that were randomly rotated between trials. The orientations of the pacman gaps were selected from 18 possible orientations (0° - 340°, in 20° steps). In Kanizsa displays, the stimuli within each configuration were aligned to form an illusory triangle. In random displays, the orientations were selected randomly with the constraint that they did not form illusory triangles.

**Fig. 1:**
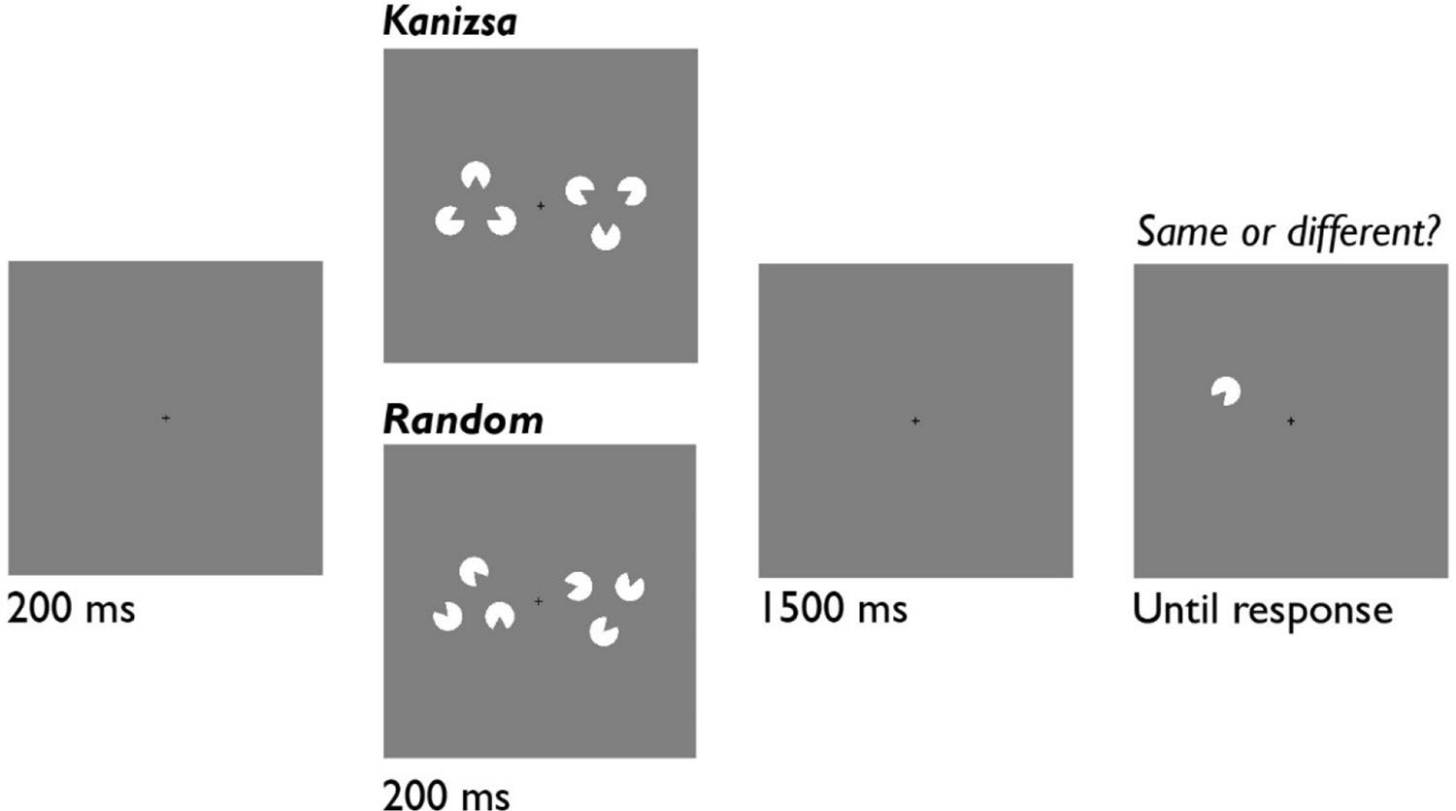
Trial Procedure for Experiment 1.* Note.* On each trial, the stimuli were either aligned to form illusory triangles (Kanizsa) or were randomly oriented (random). We manipulated the probability that Kanizsa versus random displays were presented for each block (80% vs 20%)

At the start of each trial, a fixation cross (length 0.5°) was displayed for 200 ms and remained on screen throughout the trial. The memory display was then presented for 200 ms, followed by a delay of 1500 ms. Next, the test item was presented. On 50% of trials, the test item was the same as the target that appeared at the same location. On remaining trials, the test item was replaced with an item that was not present in the memory display and was at least 40° from the target orientation. Participants pressed “Z” or “/” to indicate whether the test item and the target were same or different. Afterwards, feedback (“CORRECT” in green or “INCORRECT” in red) was displayed for 1000 ms. The following trial began after an intertrial interval of 1000 ms. Trial procedure is illustrated in Fig. 1.

We used a blocked design to examine effects of task context. Given that grouping strategies seem to be under top-down control (Magen & Berger-Mandelbaum, 2018; van Lamsweerde et al., 2016), we expect participants to use grouping cues flexibly depending on task expectations. In high-probability blocks, the majority of trials (80%) contained Kanizsa displays. In low-probability blocks, only 20% of the trials contained Kanizsa displays. Instructions at the start of each block informed participants of the block type. The task consisted of 300 trials (10 blocks). Blocks were evenly divided between high-probability and low-probability blocks. Block order was presented in strict alternation (e.g., high, low, high, etc.), and the first block was counterbalanced between participants. Twenty practice trials (ten of each probability condition) preceded the main task.

#### WM capacity task

Prior to the orientation memory task, we used a colour change detection task to measure WM capacity (Allon et al., 2019; Luck & Vogel, 1997). Memory stimuli were 4 or 8 squares (1.24° × 1.24°) whose colours were randomly selected, without replacement, from nine distinct colours (red (255, 0, 0), green (0, 255, 0), blue (0, 0, 255), yellow (255, 255, 0), magenta (255, 0, 255), cyan (0, 255, 255), white (255 255 255), black (0, 0, 0), orange (255, 128, 0)). All squares were presented within a 16.6° × 16.6° region around fixation. The center of each square had a minimum 2° horizontal distance from fixation and a minimum 2° distance from any other square.

Each trial began with the presentation of a fixation cross (1000 ms). Next, memory stimuli were presented for 150 ms. Following a delay (900 ms), a test square appeared, and participants had to press “Z” or “/” to indicate whether it was the same colour as the memory square presented at that location. On 50% of trials, the test item was a new colour that had not appeared on that trial. The following trial began after participants responded. There were 120 trials (3 blocks), preceded by 5 practice trials.

### Results and discussion

**Fig. 2:**
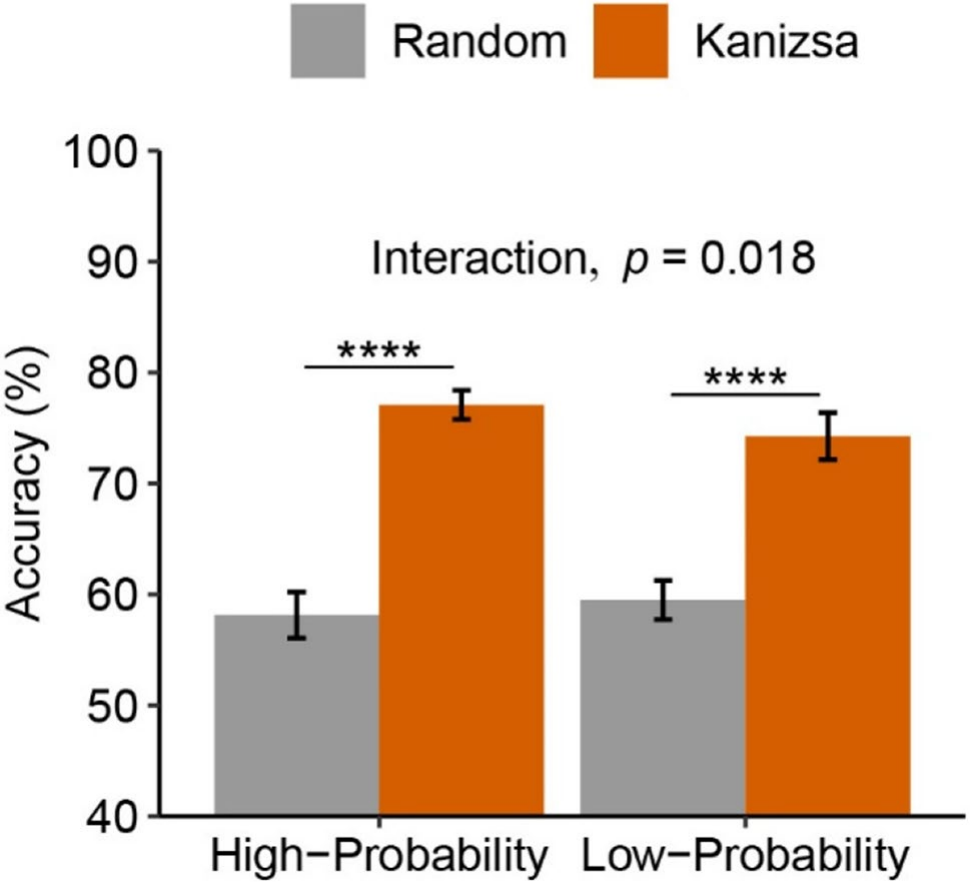
Orientation Task Accuracy in Experiment 1. *Note.* Mean accuracy as a function of block type and grouping condition. Here, high-probability and low-probability blocks refer to the probability that Kanizsa versus random displays appear in that block. Errors bars represent 95% within-subject CI (Morey, 2008)

Orientation task accuracy (see Fig. 2) was analyzed with a 2 (grouping: Kanizsa, random) × 2 (probability: high, low) ANOVA. We found higher accuracy for Kanizsa (75.68%) versus random condition (58.82%), *F*(1,49) = 238.55, *p* < .001, η_p_^2^ = .830, replicating previous work (Allon et al., 2019; Chen et al., 2021; Diaz et al., 2021)^1^. There was no difference between high-probability and low-probability blocks (67.61% vs. 66.88%), *F*(1,49) = 0.89, *p* = .352, η_p_^2^ = .018. Critically, we found a grouping × probability interaction, *F*(1,49) = 5.99, *p* = .018, η_p_^2^ = .109, showing a larger Kanizsa benefit in the high-probability versus low-probability block (18.95% vs. 14.77%). This is consistent with participants strategically using the Kanizsa figure when it is beneficial.

#### Individual differences

Table 1 provides descriptive statistics for measures that are examined in correlations.

**Table 1:** Descriptive Statistics for Main Measures

Measure	*M*	*Mdn*	*SD*	Skew	Kurtosis	Reliability
Experiment 1						
WM capacity	2.79	2.87	0.90	−0.26	0.05	.73
Overall accuracy	67.87	68.17	5.76	−0.12	−0.58	.79
Difference benefit	17.29	17.33	6.97	−0.19	−0.80	.43
Ratio benefit	0.43	0.41	0.18	−0.09	−0.53	.66
Experiment 2						
WM capacity	2.69	2.60	0.80	0.26	−0.87	.65
Overall accuracy	73.99	74.22	9.98	−0.08	−1.07	.94
Difference benefit	9.42	9.69	6.91	−0.27	−0.53	.53
Ratio benefit	0.35	0.35	0.27	0.13	−0.82	.76

Note. We computed Spearman-Brown corrected reliability estimates over 5000 random splits (Parsons et al., 2019). For WM capacity, we computed split-half reliability with the constraint that there was an equal number of set size 4 and 8 trials in each random split.

For the WM capacity task, we first computed *K* for each set size using Cowan’s (2001) formula *K* = Set size × (Hit – False alarm), and took the average to estimate a participant’s capacity. Overall, WM capacity is positively correlated with accuracy in the orientation memory task, *r* = .48, *t*(48) = 3.75, *p* < .001, 95% CI = [.23, .67].

We assessed grouping strategy in two ways. First, we computed the accuracy difference between Kanizsa and random trials (difference benefit). We chose to collapse across probability conditions since we observed large accuracy differences in both conditions. Nevertheless, one caveat of using the accuracy difference is that this measure does not consider potential ceiling effects among individuals with high WM capacity. That is, high-capacity individuals may show little benefit from the Kanizsa if they are highly accurate in the random condition. Therefore, we also computed a ratio benefit to assess participants’ improvement from random to Kanizsa condition compared to the maximum possible improvement towards ceiling performance (see also McCollough, 2011): (Kanizsa accuracy – Random accuracy)/(100 – Random accuracy).

**Fig. 3:**
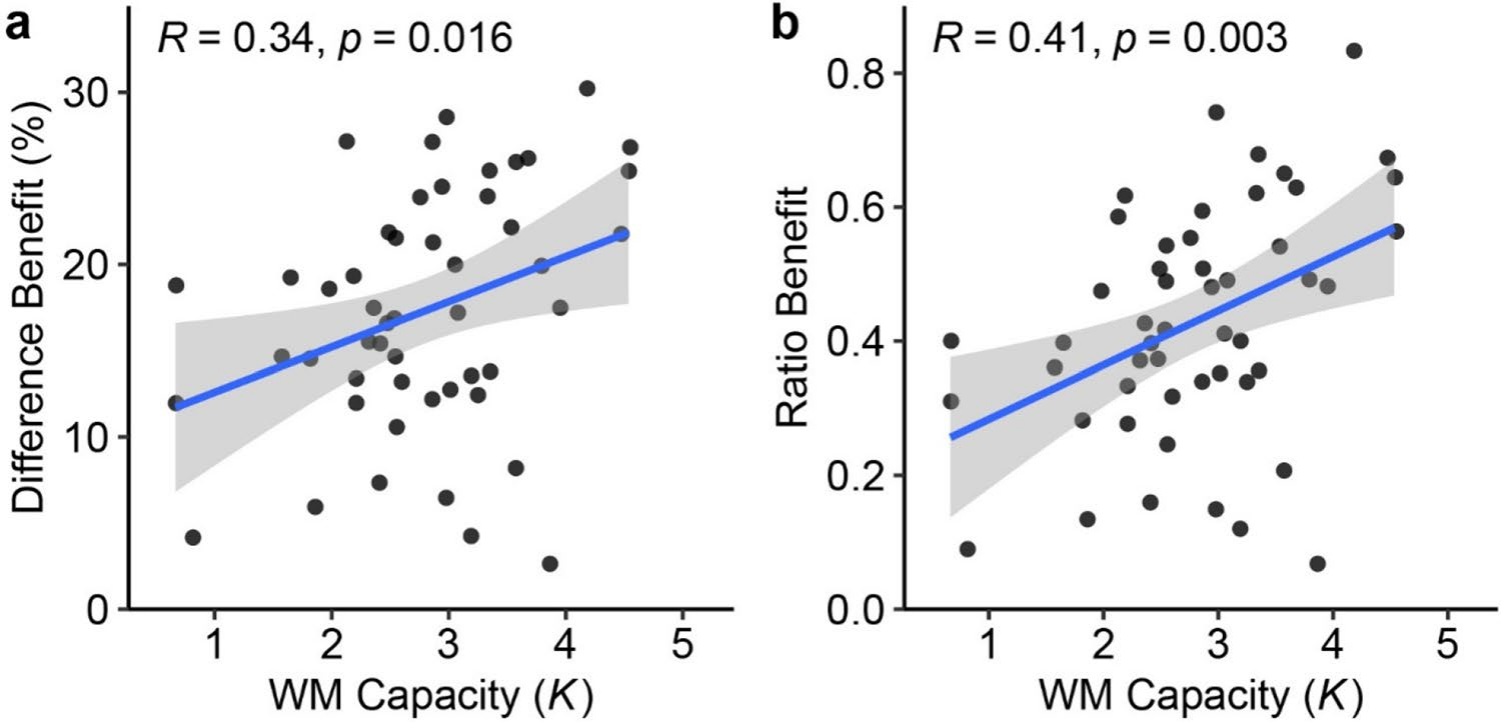
Individual Differences in Experiment 1. *Note.* Scatterplots with best-fitting regression line showing (**a**) the correlation between WM capacity and the difference benefit and (**b**) the correlation between WM capacity and the ratio benefit

We computed both the observed correlation and the disattenuated correlation, which corrects for imperfect reliability and provides an estimate for the true relationship (Spearman, 1904). We found higher WM capacity predicted a larger difference benefit (see Fig. 3), *r* = .34, disattenuated *r* = .60, *t*(48) = 2.49, *p* = .016, 95% CI = [.07, .56]. Similarly, WM capacity was positively correlated with the ratio benefit, *r* = .41, disattenuated *r* = .59, *t*(48) = 3.14, *p* = .003, 95% CI = [.15, .62]. Taken together, these findings suggest that higher WM capacity is related to more efficient use of grouping strategy.^2^

## Experiment 2

Experiment 1 provided evidence that higher WM capacity predicts more effective use of grouping strategy. However, these individual differences could reflect differences in low-level perceptual abilities relating to the sensory integration of the illusory information. Critically, studies have suggested low-level sensory processing of the Kanizsa object is not necessary for a WM benefit, such as when the inducing pacmen are sequentially presented (Gao et al., 2016; Zhang & Du, 2022). Moreover, work has shown a memory benefit for pacman stimuli that face away from the center of the configuration (Chen et al., 2021). These findings suggest that participants are able to use more abstract rules to encode and group items.

Here we assessed the degree to which individuals use more conceptual forms of grouping to improve performance. Specifically, we sequentially presented items that contained random orientations or were aligned to form Kanizsa figures. If individual differences in grouping indeed relate to strategic control processes, we should still observe a positive relationship between WM ability and grouping benefits. In addition, we reasoned that changing to a sequential presentation format would make it more difficult to apply grouping strategies. Therefore, in Experiment 2, we reduced the memory set size and used Kanizsa rectangles instead of triangles. We also removed the probability manipulation since we observed large grouping benefits in both low- and high-probability conditions in Experiment 1.

### Method

#### Participants

Fifty new participants (33 women, 17 men; mean age = 19.06 years, age range =18 –29) took part in the experiment for course credit.

Twelve additional participants were excluded: 6 did not perform reliably above chance for the orientation task (below 54.41%), 3 completed fewer than 80% trials due to a computer code error, 1 withdrew from the study, 1 did not complete the WM capacity task, and 1 reported colour vision deficiency.

#### Apparatus

The study was run on a Dell OptiPlex 7000 computer and a 24-inch Dell G2422HS monitor (60 Hz refresh rate, 1,920 × 1,080 pixels).

#### Procedure

As in Experiment 1, participants completed the WM capacity task prior to the orientation memory task.

In the orientation memory task, the stimuli were four pacmen (1° radius, with rectangular gaps) arranged in two spatially proximal pairs. Stimuli in the same pair were 4° apart, and stimuli in different pairs were at least 6° away. We manipulated whether the pacman gaps for each pair were aligned (Kanizsa vs. random). Critically, we sequentially presented the stimuli in two displays, and items in the same pair were never presented at the same time. Participants were explicitly informed of the grouping manipulation and were shown examples of Kanizsa and random displays.

**Fig. 4:**
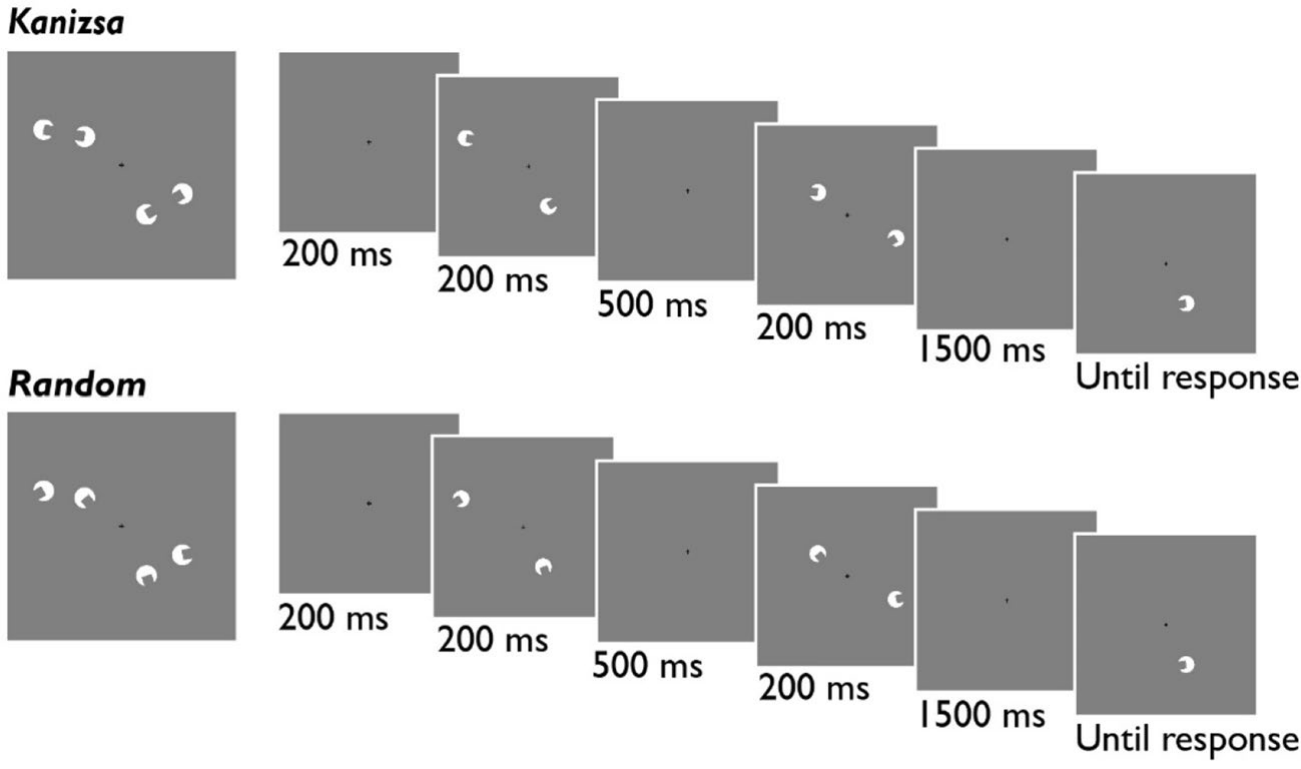
Trial Procedure for Experiment 2. *Note*. Stimuli consisted of two pairs of pacman stimuli. We manipulated whether the pacman gaps in each pair were aligned. On each trial, the stimuli were sequentially presented such that only one item from each pair were presented at a time.

Each trial began with a fixation cross (200 ms). Next, the first display appeared on the screen for 200 ms. After a short delay (500 ms), the second display was presented for 200 ms. Following another delay (1500 ms), the test item was presented. As in Experiment 1, participants responded whether the test item was same or different from what they memorized. Trial procedure is shown in Fig. 4.

The task contained 320 trials (10 blocks), preceded by 20 practice trials. Within each block, trials were evenly divided between 2 grouping (Kanizsa, random) × 2 display tested (first, second) conditions.

### Results and discussion

**Fig. 5:**
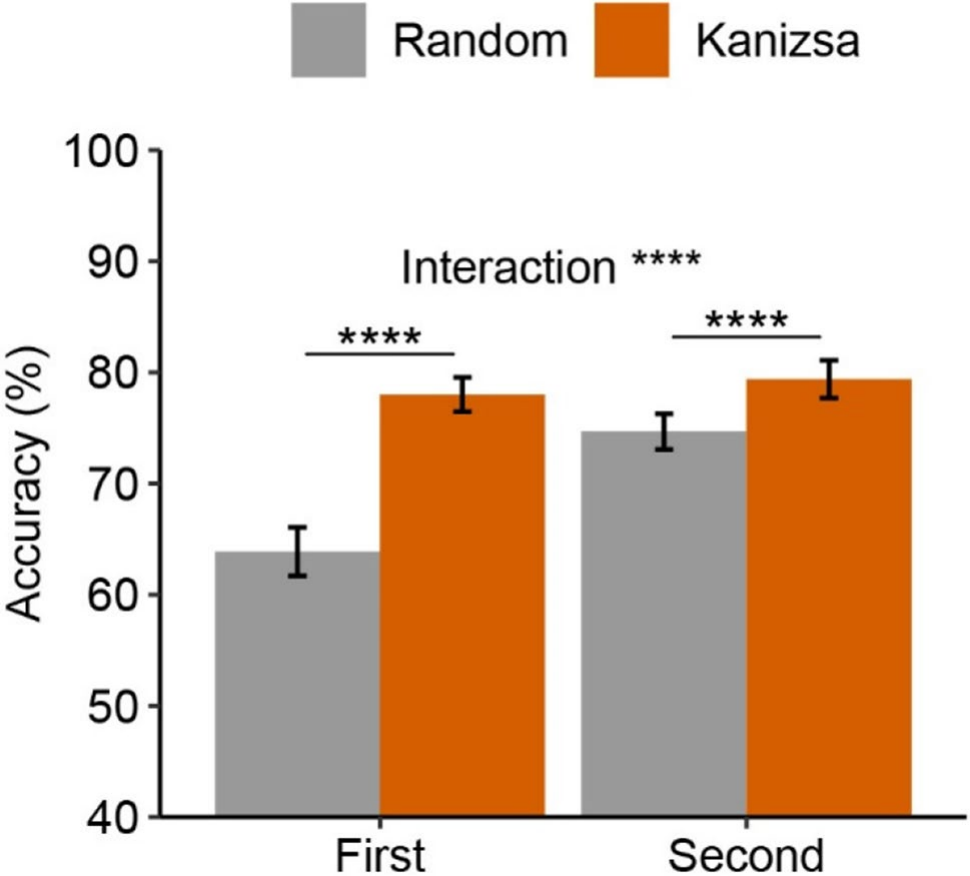
Orientation Task Accuracy in Experiment 2. *Note.* Mean accuracy as a function of display tested (first, second) and grouping condition. Errors bars represent 95% within-subject CI (Morey, 2008)

We analyzed orientation task accuracy using a 2 (grouping: Kanizsa, random) × 2 (display tested: first, second) ANOVA (see Fig. 5). We examined effects of sequential order to better understand how participants integrate items across the two displays.

Results showed higher accuracy for Kanizsa (78.70%) versus random conditions (69.27%), *F*(1,49) = 92.95, *p* < .001, η_p_^2^ = .655. This replicates studies showing a benefit for Kanizsa figures when items are sequentially presented (Gao et al., 2016; Zhang & Du, 2022). Overall, there was better performance when the tested item was from the second display (77.03%) versus the first display (70.94%), *F*(1,49) = 46.00, *p* < .001, η_p_^2^ = .484. We found a grouping × display interaction, *F*(1,49) = 37.67, *p* = < .001, η_p_^2^ = .435. Participants showed a larger Kanizsa benefit when they were tested on the first display (14.13%) compared to the second display (4.72%). This suggests that participants are making use of their knowledge about the grouping manipulation to rescue information that has already been encoded.

#### Individual differences

Descriptive statistics are listed in Table 1. Overall, higher WM capacity is associated with higher accuracy in the orientation memory task, *r* = .60, *t*(48) = 5.24, *p* < .001, 95% CI = [.39, .76].

**Fig. 6:**
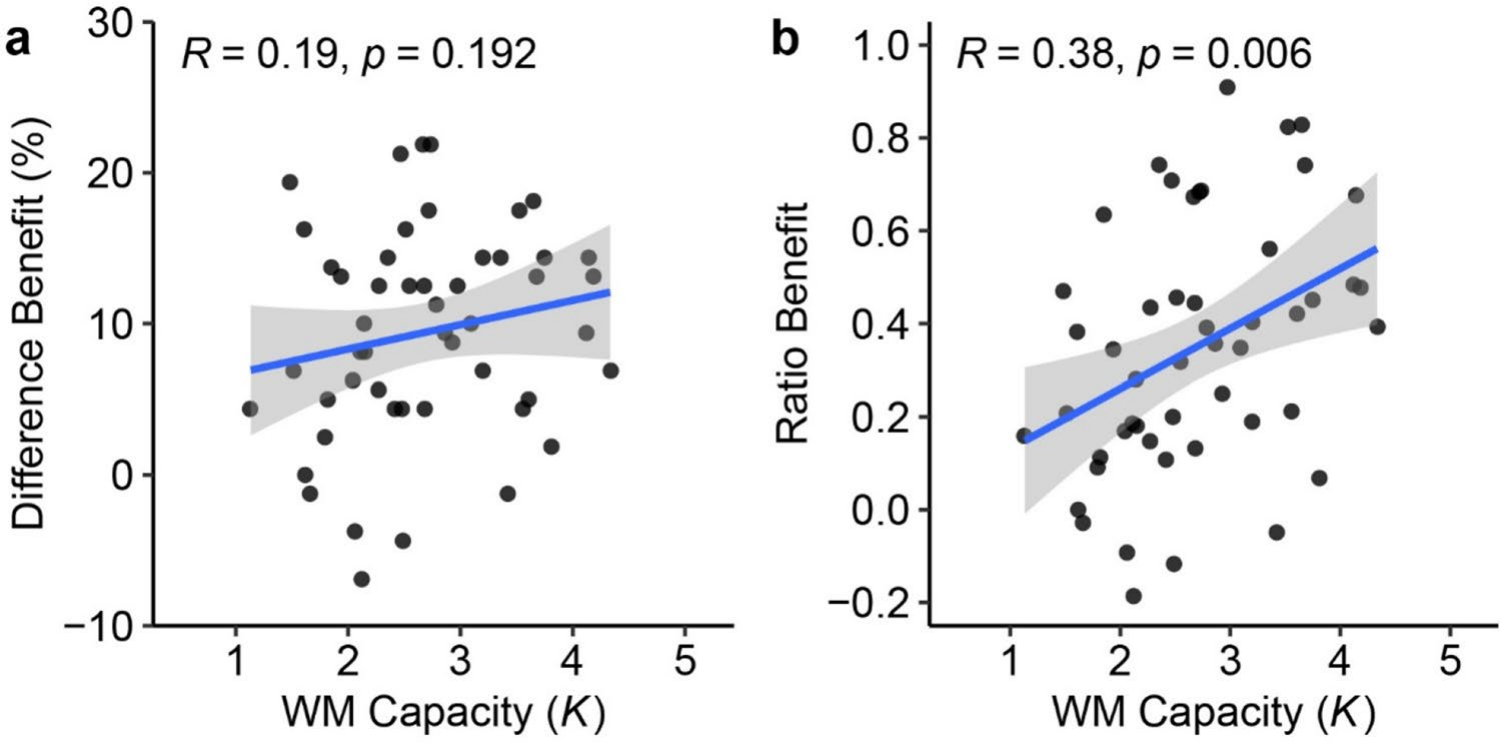
Individual Differences in Experiment 2. *Note.* Scatterplots with best-fitting regression line showing (**a**) the correlation between WM capacity and the difference benefit (**b**) the correlation between WM capacity and the ratio benefit

As in Experiment 1, we computed both difference benefits and ratio benefits to assess strategic use of grouping (see Fig. 6). Results showed that WM capacity is not related to the difference benefit, *r* = .19, disattenuated *r* = .32, *t*(48) = 1.32, *p* = .192, 95% CI = [-.10, .44]. However, we did find that WM capacity is positively correlated with the ratio benefit, *r* = .38, disattenuated *r* = .55, *t*(48) = 2.89, *p* = .006, 95% CI = [.12, .60]. Although the pattern of results was less clear-cut than Experiment 1, these findings provides some evidence that WM capacity is related to grouping based on more high-level knowledge. It is interesting that we found different results when using the difference benefit and the ratio benefit. We noted that in both experiments the ratio benefit has higher reliability estimates than the difference benefit. In fact, previous work has suggested that difference score metrics have low reliability (Hedge et al., 2018). We speculate that poor reliability in difference scores may have attenuated the observed relationship. Moreover, another limitation of this study is that the sample size may not provide sufficient power for detecting smaller correlations.

### Discussion

Visual WM is limited, but we can use strategies to boost performance. To what extent does strategy use vary across individuals? Here we examined whether WM capacity is related to the use of grouping strategy. In Experiment 1, we found that individuals with higher WM capacity showed a larger benefit in remembering Kanizsa figures. Further, Experiment 2 suggested that these individual differences may partially reflect a more abstract grouping mechanism: Individuals with higher WM capacity also seem to show more efficient grouping under sequential encoding. 

Previous work suggests that individual differences in WM may relate to differences in ability and/or strategy (Gonthier, 2021; Pearson & Keogh, 2019). Our results show one way that ability and strategy are related. This could suggest that individuals with higher WM ability have more resources to use more demanding strategies (Turley-Ames & Whitfield, 2003). Alternatively, it may be that capacity estimates from the change detection task are not pure measures of WM ability, but they may partially reflect differences in strategy use (e.g., Bengson & Luck, 2016). For example, work has suggested individuals have better WM performance when attempting to encode everything in the display instead of encoding only a subset of the information (Bengson & Luck, 2016; Wang et al., 2020; but see Atkinson et al., 2018; Cusack et al., 2009; Linke et al., 2011). Moreover, WM performance may reflect differences in how individuals make use of ensemble encoding and chunking strategies (Brady & Alvarez, 2011; Haberman et al., 2015; Nassar et al., 2018). Future work is necessary to examine how WM ability relates to a wider range of strategies. Furthermore, it is likely that the use of grouping strategy has influenced various processes in WM, including encoding, storage, and updating of information. Further work is needed to better understand how strategy use modulates these processes.

Here we found memory benefits both when Kanizsa figures are simultaneously and sequentially presented. Therefore, the observed differences in strategy use may partly reflect differences in the ability to parse visual information into illusory shapes in limited time and/or differences in strategic grouping of information into meaningful units. Moreover, we chose to explicitly inform participants about the grouping manipulation to maximize the chances of finding large memory benefits. Thus, there could be differences in how individuals use prior knowledge when encoding and storing information. Furthermore, previous EEG studies have suggested that Kanizsa figures improve performance by reducing neural resources required to maintain the items (Diaz et al., 2021; McCollough, 2011; but see Heisterberg, 2021). Others have proposed that Kanizsa figures enhance tagging and encoding of information (Allon et al., 2019). Future work using EEG could be helpful in understanding mechanisms behind the Kanizsa benefit as well as potential differences in how individuals represent Kanizsa figures in WM.

## Supplementary information

Below is the link to the electronic supplementary material.

Supplementary file1 (DOCX 1617 KB)

## Data Availability

Data are available on the Open Science Framework (https://osf.io/fvq9x/).

## Footnotes

1 We further conducted exploratory analyses to assess whether Kanizsa benefit may in part reflect strategic guessing. These analyses are presented in the supplemental materials.

2 One might question whether the relationship between WM capacity and Kanizsa benefit results from more efficient use of strategy or lack of effort in the random condition. We report analysis in the Supplemental Materials examining whether individuals with higher WM capacity may have put less effort into the random condition.

## Code availability

Experiment code and analysis scripts can be accessed at https://osf.io/fvq9x/.
