# Supplementary material for: Assessing individual differences in grouping strategy in visual working memory
Source: Atten Percept Psychophys. 2025 Feb 26;87(3):728–36. doi: 10.3758/s13414-025-03013-w (PMC11965235; doi:10.3758/s13414-025-03013-w)
Supplement: Supplementary file 1 — Supplementary file1 (DOCX 1617 KB) [file 13414_2025_3013_MOESM1_ESM.docx]

# Supplemental Materials

1. **Guessing strategy**

Our study found a memory benefit for items that formed Kanizsa figures versus items that were randomly oriented. This is consistent with the idea that participants are using grouping strategies to improve performance. However, it is possible that the Kanizsa benefit in part reflects strategic guessing.

Here we examined the possible use of guessing strategies in Experiment 1. In our first analysis, we simply wanted to see if the overall pattern of responses is influenced by participants’ knowledge of Kanizsa pacman locations and orientations. Pacmen near the border must have their gap facing away from the border to plausibly allow for all 3 items to fit in the display. Second, since we used a “protection zone” around fixation (which participants may or may not have learned), pacmen near the center of the screen cannot face toward the fixation point. To examine this, we first divided trials into different spatial bins based on a tertiary split of the absolute horizontal and vertical distance between the test item and the fixation (see Figure S1). For each spatial bin, we ran a logistic regression model using individual trial responses (1 = same, 0 = different) as the outcome variable. The predictor variable is the degree to which the pacman gap faces toward or away from the fixation, defining 0° as the angle in which the gap is directly facing fixation and greater values representing the absolute value of the pacman’s angular rotation away from fixation (with 180° facing fully away).

**Figure S1**

Schematic of Spatial Bins


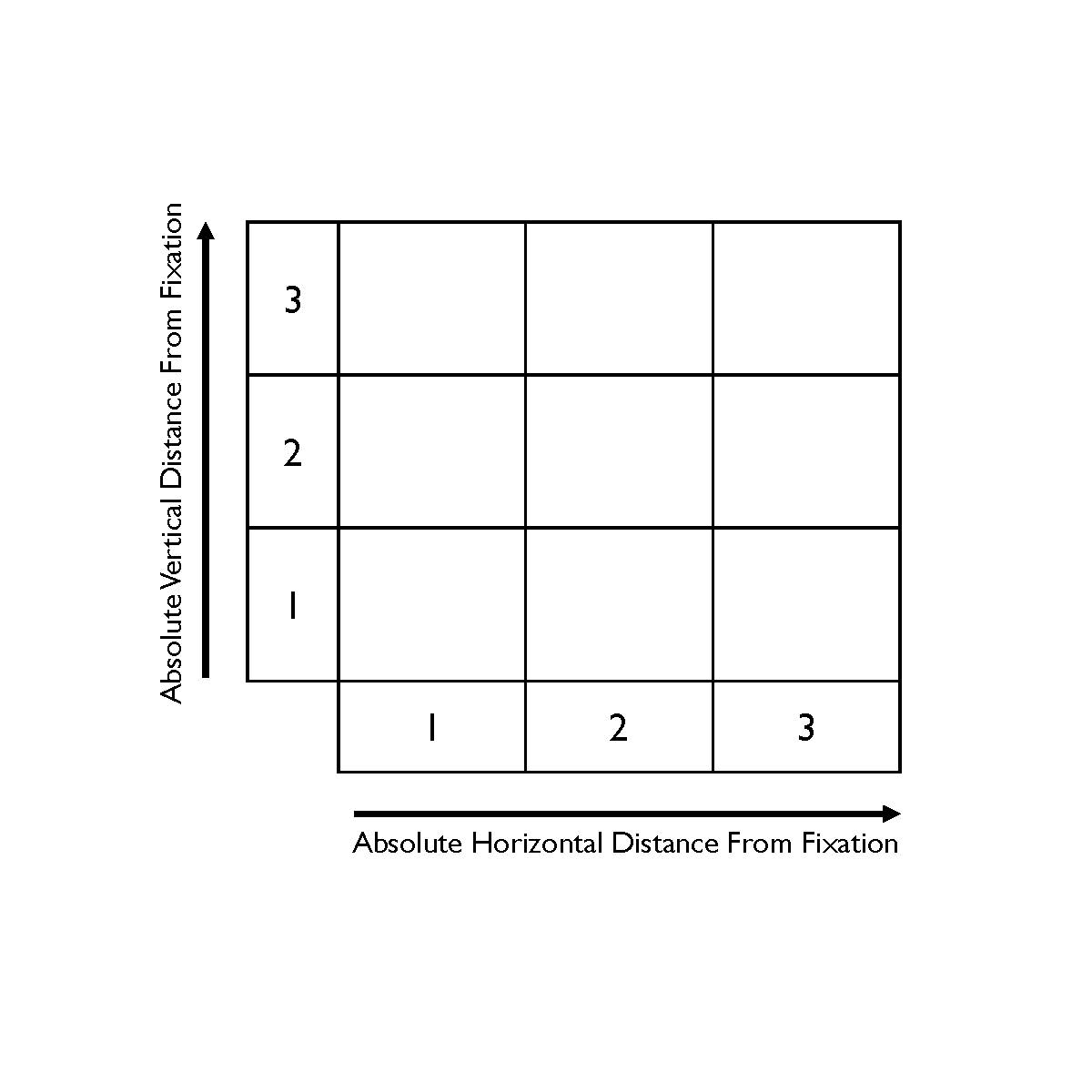


*Note.* We divided trials into spatial bins using a tertiary split of the absolute horizontal and vertical distance between the test item and the fixation cross (1 = closest to fixation, 3 = furthest away from fixation).

On Kanizsa trials, we found that when the test pacman was near the border, participants were considerably more likely to respond “same” if the pacman gap faced toward the fixation point. When the test pacman was near the fixation, participants were more likely to respond “same” if the pacman gap faced away from the fixation point (Figure S2). Note that it is not possible for us to determine whether this pattern is due solely to participants simply remembering the correct orientation vs. using strategic guessing. However, when we analyzed the pattern of responses in the random condition, the biases were largely reduced (Figure S3). This shows that any guessing pattern that may have been used on Kanizsa trials was not extended to the random trials.

**Figure S2**

*Response Patterns in the Kanizsa Condition in Experiment 1*

*Note.* Scatterplots showing the relationship between individual trial responses and the degree to which the test item faces the fixation. The blue lines show the best-fitting logistic regression lines.


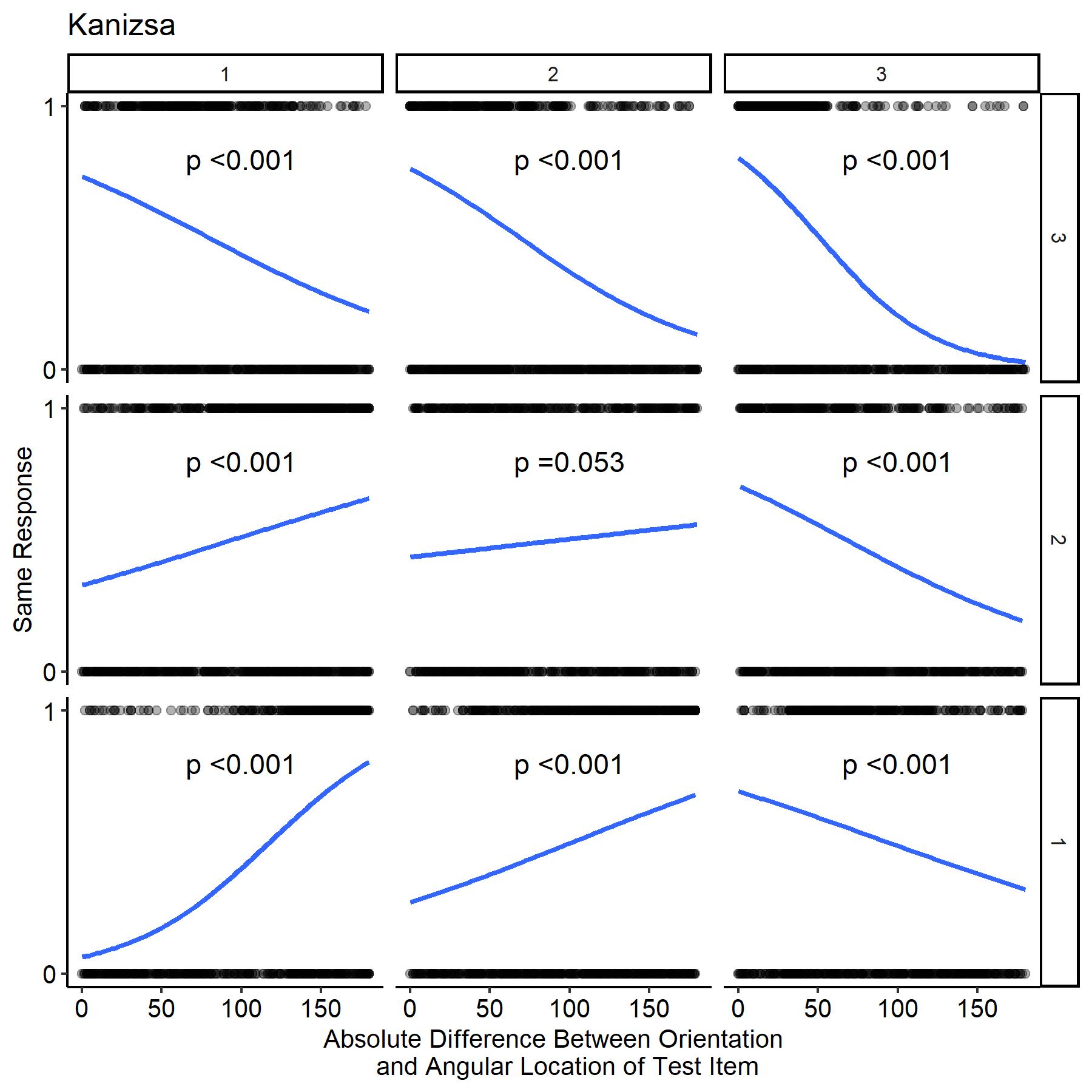


**Figure S3**

*Response Patterns in the Random Condition in Experiment 1*

*Note.* Scatterplots showing the relationship between individual trial responses and the degree to which the test item faces the fixation. The blue lines show the best-fitting logistic regression lines.


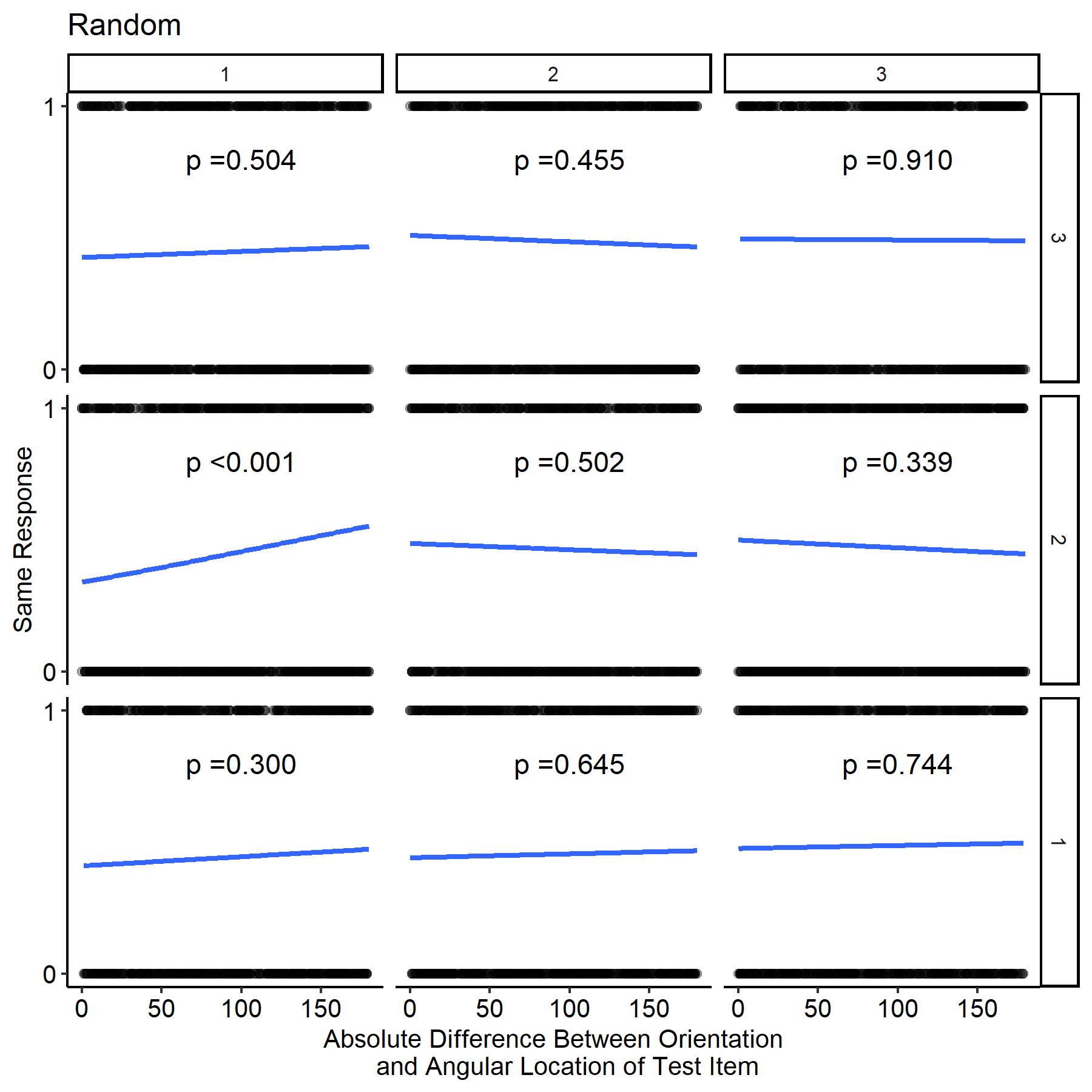


The next analysis attempted a more direct test of guessing strategy on Kanizsa trials. On these trials, participants may only remember the location of items but not the orientation of items. They could then use the knowledge that the pacman gaps face inward in Kanizsa triangles to infer the angle of each pacman shape. If this is the case, participants should be more likely to respond “same” (less likely to respond “different”) when the test item conforms to a Kanizsa triangle. To examine this, using Experiment 1 data, we analyzed participants’ hit rate (correct response to different test pacman) and false alarm (incorrect response to same test pacman) for random trials. We found that participants were less likely to respond “different” when the test item conforms to a Kanizsa triangle (see Figure S4). This indicates that participants did indeed bias their guessing strategy based on the expectancy of a Kanizsa trial. As a result, it appears that at least some of the Kanizsa benefit observed in the present study is due to strategic guessing.

**Figure S4**

*Hit Rate and False Alarm in the Random Condition in Experiment 1*


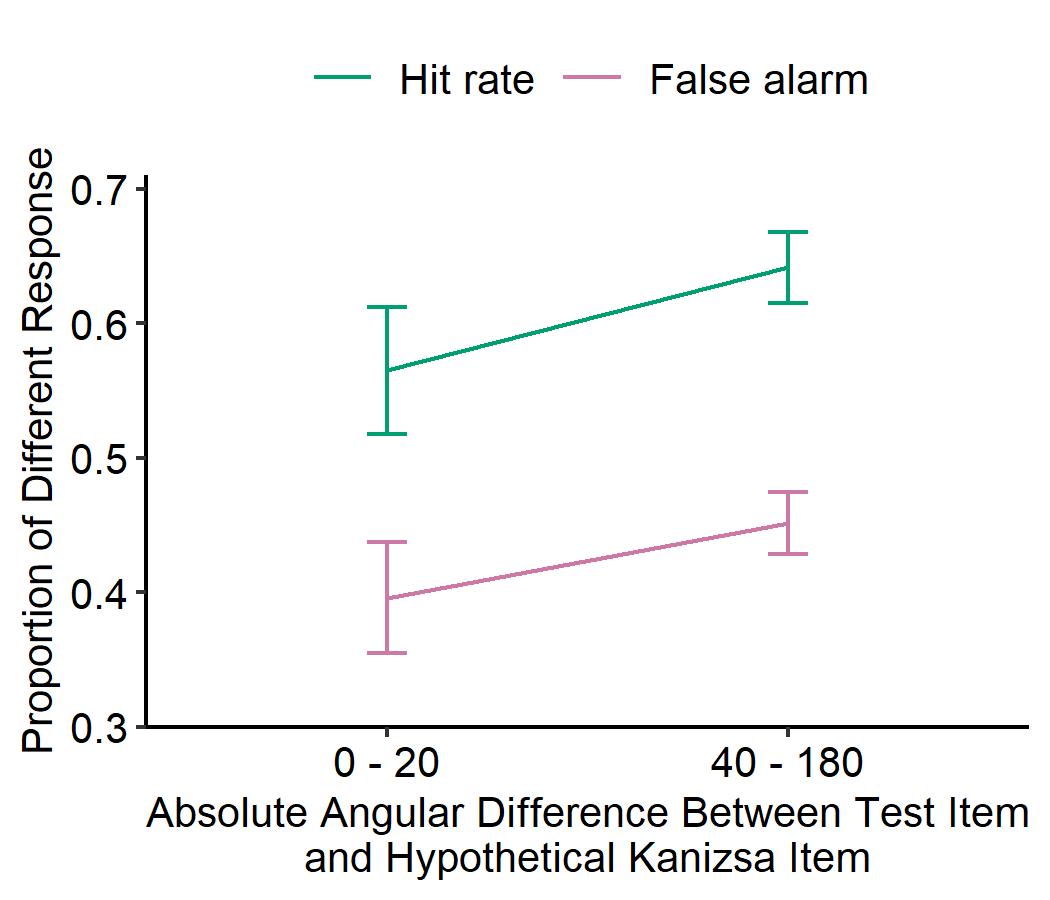


*Note.* We assessed whether responses in the random condition depend on whether the test item conforms to a Kanizsa triangle. Errors bars represent within-subject 95% CI (Morey, 2008).

1. **Is the Kanizsa benefit explained by lack of effort in the random condition?**

Here we conducted exploratory analysis to examine whether individuals with higher WM capacity may have put less effort into the random condition, resulting in a larger grouping benefit in these individuals. If individuals with high WM capacity did not try as hard to encode information in the random condition, we would expect these individuals to have worse performance on random trials in high-probability blocks, where they expect more Kanizsa trials. To examine this, we divided participants from Experiment 1 into high and low-capacity groups using a median split of their WM capacity estimate (*Mdn* = 2.87). Memory accuracy on proximity trials was numerically lower in high-probability blocks compared to low-probability blocks for both high-capacity and low-capacity groups. However, the accuracy difference between high-probability and low-probability blocks did not differ across groups (1.31 vs. 1.42), *t*(48) = 0.04, *d_s_* = .01, *p* = .966 (see Figure S5).

**Figure S5**


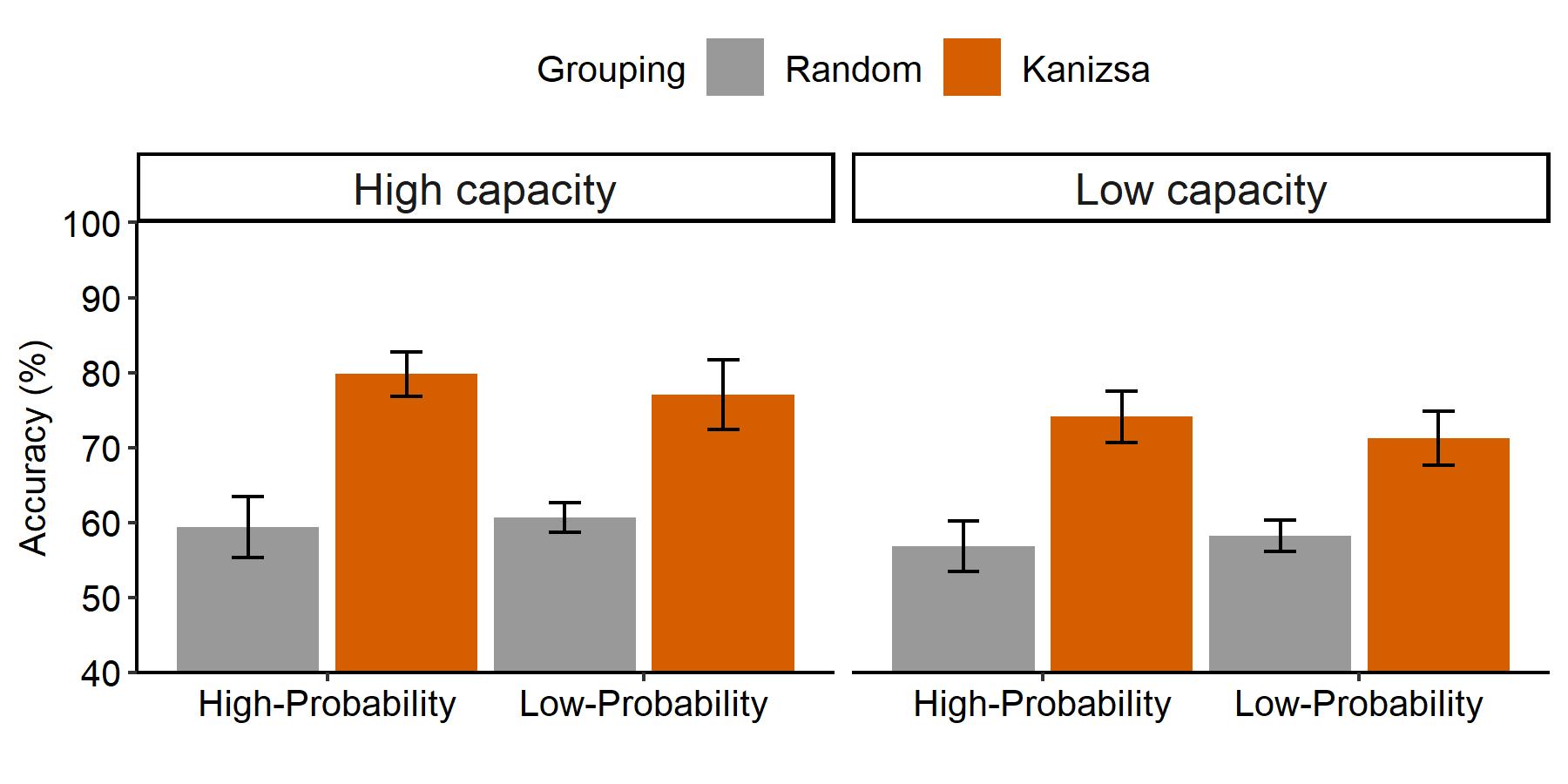
*Orientation Task Accuracy Across Capacity Groups in Experiment 1*

*Note.* We used median split to divide participants into high and low-capacity groups. Errors bars represent 95% CI.
